# Fever burden within 24 h after hematoma evacuation predicts early neurological deterioration in patients with intracerebral hemorrhage: a retrospective analysis

**DOI:** 10.3389/fneur.2023.1205031

**Published:** 2023-07-19

**Authors:** Fan Wu, Yu Xiong, Shi-ling He, Xiao-hua Wang, Xin-li Chen, Wei-can Chen, Qiao-mei Huang, Xin-yue Huang, Zhi-gang Pan, Wei-peng Hu, He-fan He, Feng Zheng

**Affiliations:** ^1^Department of Anesthesiology, The Second Affiliated Hospital of Fujian Medical University, Quanzhou, China; ^2^Department of Neurosurgery, The Second Affiliated Hospital of Fujian Medical University, Quanzhou, China

**Keywords:** intracerebral hemorrhage, hematoma evacuation, fever burden, early neurological deterioration, body temperature, duration of fever

## Abstract

**Background:**

Early neurological deterioration after hematoma evacuation is closely associated with a poor prognosis in patients with intracerebral hemorrhage. However, the relationship between body temperature after hematoma evacuation and early neurological deterioration remains unclear. Therefore, this study aims to explore the possible relationship between body temperature and early neurological deterioration in patients with intracerebral hemorrhage after hematoma evacuation.

**Methods:**

We retrospectively collected data from patients with cerebral hemorrhage at our institute between January 2017 and April 2022. The Student’s *t*-test, Mann–Whitney U-test, and χ^2^ Test and Fisher’s exact test were used to analyze the clinical baseline data. A univariate logistic regression model was used to evaluate the association between the body temperature indices and early neurological deterioration. The predictive power was assessed using the area under the Receiver Operating Characteristic (ROC) curve. The secondary outcome was a poor functional outcome.

**Results:**

Among 2,726 patients with intracerebral hemorrhage, 308 who underwent hematoma evacuation were included in the present analysis. A total of 82 patients (22.6%) developed early neurological deterioration. Univariate analysis showed that sex (*p* = 0.041); body temperature at 6 h (*p* = 0.005), 12 h (*p* = 0.01), and 24 h (*p* = 0.008) after surgery; duration of fever (*p* = 0.008); and fever burden (*p* < 0.001) were associated with early neurological deterioration. Multivariate logistic regression showed that fever burden was independently associated with early neurological deterioration (OR = 1.055 per °C × hour, 95%CI 1.008–1.103, *p* = 0.020). ROC showed that fever burden (AUC = 0.590; 95%CI: 0.514–0.666) could predict the occurrence of early neurological deterioration.

**Conclusion:**

Fever burden is associated with early neurological deterioration in intracerebral hemorrhage patients undergoing hematoma evacuation. Our findings add to previous evidence on the relationship between the fever burden and the occurrence of early neurological deterioration in patients with intracerebral hemorrhage. Future studies with larger sample sizes are required to confirm these findings.

## 1. Introduction

Intracerebral hemorrhage (ICH) is a stroke subtype with high morbidity and mortality (1). For patients with spontaneous superficial cerebral hemorrhage without intraventricular hemorrhage, early surgical intervention may confer a clinically relevant survival advantage (2). Owing to its poor prognosis, many researchers are dedicated to optimizing the treatment strategy for ICH (3–5). Early neurological deterioration (END), with an incidence of approximately 25.4%, is considered to be associated with poor prognosis among patients with ICH (6, 7). Therefore, identifying high-risk stroke patients with END is crucial for neurologists. The current methods for identifying high-risk individuals depend largely on the assessment of the clinical and neuroimaging features present at the time of onset. In recent years, researchers have focused more on END in ischemic stroke (8–10). It was reported that the maximum body temperature and fever burden within 24 h after endovascular thrombectomy were independent predictors of END in ischemic stroke patients with large vessel occlusion (11). Fever occurs in approximately 40% of patients with ICH and is independently associated with a poor outcome and increased mortality (8). Therefore, the management of fever in patients with cerebral hemorrhage is particularly important. Recently, the INTERACT-3 trail findings revealed that the implementation of the care bundle procedure, including intensive blood pressure lowering, strict glucose control, antipyrexia treatment and rapid reversal of warfarin-related anticoagulation within a few hours after the onset of symptoms can improve the functional outcome of patients with acute cerebral hemorrhage (12). Previous studies have confirmed that the body temperature is associated with END in patients with ischemic stroke after endovascular thrombectomy. Moreover, Leira et al. (6) found that a body temperature of >37.5°C was an independent predictor of END in patients with ICH. However, it remains unclear whether fever is associated with END in patients with ICH who are undergoing surgery. Therefore, this study explored the possible relationship between body temperature and END in patients with ICH after hematoma evacuation (HE).

## 2. Materials and methods

### 2.1. Design and patients

We retrospectively collected data from a single-center longitudinal database of patients with ICH who underwent HE at the Second Affiliated Hospital of Fujian Medical University between January 2017 and April 2022. The patients were sent to the emergency department after the onset of symptoms and then diagnosed as having cerebral hemorrhage by emergency computed tomography. Subsequently, craniotomy or endoscopic hematoma evacuation was performed immediately in patients with intracranial mass effect. The inclusion criteria were as follows: (1) diagnosis of primary ICH using computed tomography, (2) age > 18 years, and (3) HE was performed after admission. We excluded patients with secondary ICH, including traumatic brain injury, tumors and other central nervous system diseases; hemorrhagic transformation to ischemic infarction; Glasgow Coma Scale (GCS) <5 points; coagulation dysfunction; and subarachnoid hemorrhage were excluded. This study was approved by the Ethics Committee of The Second Affiliated Hospital, Fujian Medical University (IRB approval number: 2022-528) and was performed in accordance with the latest version of the Declaration of Helsinki. Due to the retrospective and observational nature of the study, the need for informed consent was waived.

### 2.2. Clinical assessment and data collection

We collected and evaluated the clinical signs and laboratory test results of the patients on admission. The Glasgow Coma Scale (GCS) was used to evaluate the severity of patients’ disturbance of consciousness, which includes three items: eye-opening, motor, and verbal responses (13). Neuroimaging examinations, including hematoma location, hematoma volume and intraventricular hemorrhage, were performed by a senior neuroradiologist with 11 years of experience. The hematoma volume was estimated using (ABC)/2 score (A and B represent the two perpendicular diameters of the largest hematoma on CT and C represents the height of the hematoma perpendicular to A and B) (14). We measured axillary body temperature using a mercury thermometer at admission and 6 h, 12 h, and 24 h after hospitalization. If the body temperature was measured more than once within the range of each time point, the highest temperature was extracted. Patients were categorized into the fever group if their body temperature was >37.5°C at any time within 24 h after surgery. To quantify the effect of fever duration and body temperature, fever burden was defined as the area on the temperature sheet that exceeded the 37.5°C threshold (11).

### 2.3. Fever management

As per our institutional guidelines and the existing literature on temperature management (14–16), we have implemented procedures for patients who develop a fever post-surgery. For patients with body temperatures below 38.3°C, we deploy physical cooling techniques, such as the use of hydropathic compresses. For patients who experience body temperatures exceeding 38.3°C, medical interventions are implemented. This typically includes an oral dose of 1 g paracetamol or an intravenous administration of 2 g metamizole, administered every 8 h.

### 2.4. Clinical outcome

The primary outcome was END, defined as a decrease in the GCS score by ≥2 points within 24 h after surgery compared to that at admission (13, 15). We also conducted modified Rankin Scale (mRS) score assessments via face-to-face follow-up or telephone interviews. The secondary outcome was poor functional outcome, defined as an mRS score greater than 2 at 3 months after discharge (16).

### 2.5. Statistical analyses

Normally distributed data were reported as mean ± standard deviation, and non-normally distributed data were reported as median (interquartile range). Categorical variables are reported as numbers (percentages). Student’s *t*-test, the Whitney U-test, χ^2^ Test, or Fisher’s exact test were used to compare variables. A binary logistic regression model was used to evaluate the relationship between variables and outcomes. Factors with *p* value less than 0.1 in univariate analysis, and other potential confounding factors were included into multivariate logistic regression analysis. The area under the ROC curve was used to calculate the predictive ability of these thermometric indices for END in patients with ICH. Sensitivity, specificity, negative predictive value (NPV), and positive predictive value (PPV) were calculated using the ROC curve. The missing data was filled by the median filling method. Statistical analyses were performed using IBM SPSS 25.0 (IBM Corp., Armonk, NY, United States). A two-tailed *p* < 0.05 was considered statistically significant.

## 3. Results

**Figure 1:**
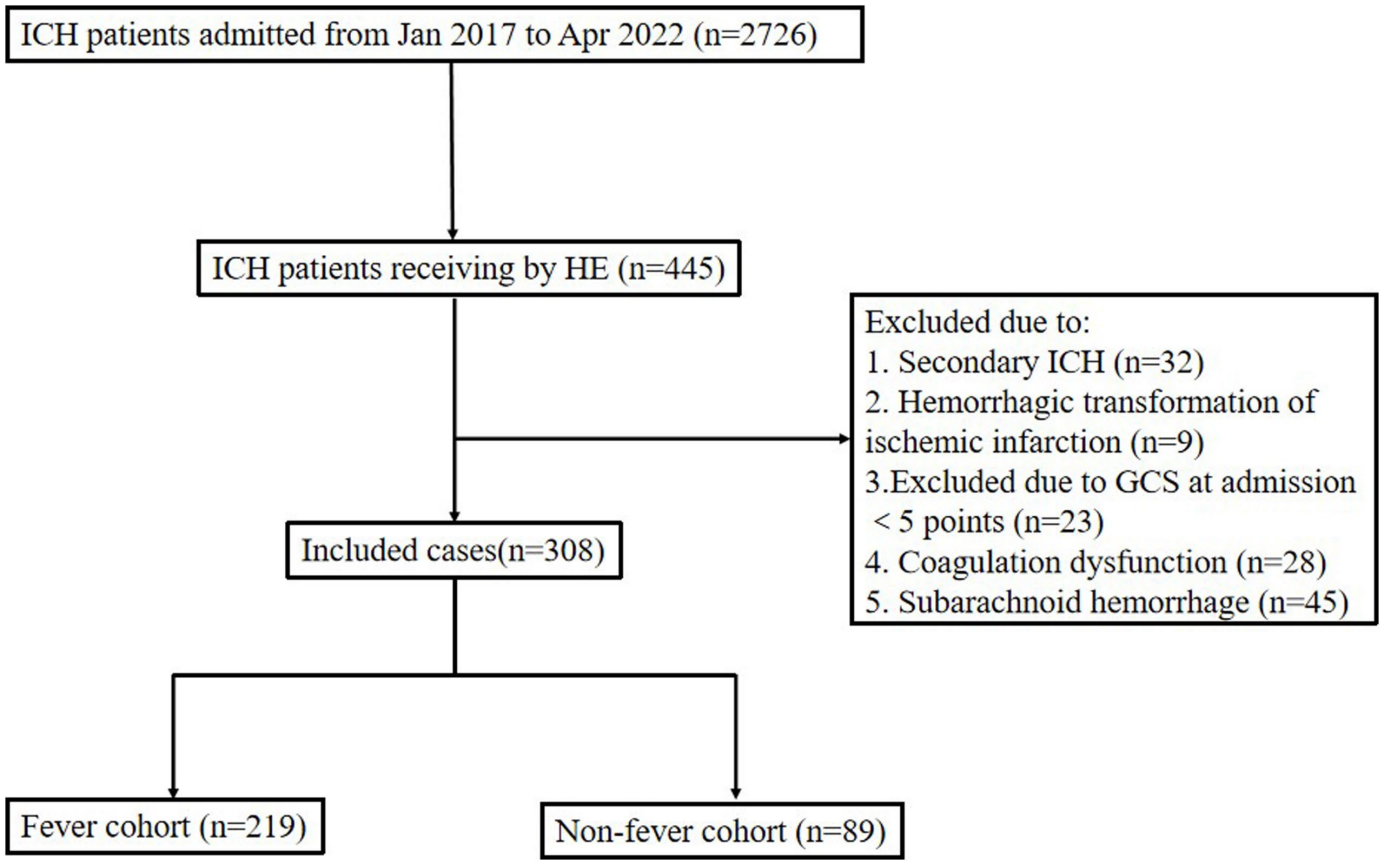
Flow chart of patient profile. ICH, intracerebral hemorrhage; HE, hematoma evacuation; GCS, Glasgow coma score.

Between January 2017 and April 2022, 2,726 patients with spontaneous cerebral hemorrhage were admitted to the Second Affiliated Hospital of Fujian Medical University. We identified 372 patients with ICH who had undergone HE. Among them, 32 patients were excluded due to secondary ICH, 9 patients were excluded due to hemorrhagic transformation of ischemic infarction, and 23 patients were excluded due to a GCS score of less than 5 at admission. Finally, 308 patients were included in the analysis (As shown in Figure 1). Twelve patients were lost to follow-up, since it did not affect the evaluation of the in-hospital outcomes; therefore, we included these 12 patients in the study. Fever occurred in 219 patients (71.1%) within 24 h post-operation. The baseline data and clinical outcomes of the patients are shown in Table 1.

**Table 1:** Baseline and clinical characteristics of patients with and without fever within 24 h after hematoma evacuation.

	Non-fever group (*n* = 89)	Fever group (*n* = 219)	*p* value
Age, y, median (IQR) (y)	55 (50–64)	51 (43–60)	**0.005**
Female sex, *n* (%)	35 (39.3)	56 (25.6)	**0.016**
GCS at admission, median (IQR)	8 (6–11)	7 (6–10)	0.083
Systolic blood pressure at admission, median (IQR), (mm Hg)	175 (158–189)	177 (154–198)	0.419
Diastolic blood pressure at admission, median (IQR), (mm Hg)	95 (86–109)	100 (85–112)	0.114
Heart rate at admission, median (IQR)	78 (70–90)	84 (75–90)	0.174
Hypertension	70 (78.7)	162 (74.0)	0.388
**Laboratory tests, median (IQR)**			
WBC × 109/L	8.13 (6.19–10.29)	8.80 (6.83–10.82)	**0.007**
Serum glucose, mmol/L	6.93 (6.30–8.11)	8.00 (6.75–9.90)	0.069
**Hematoma profiles**			
Hematoma location, *n* (%)			0.247
Basal ganglia	55 (61.8)	139 (63.5)	
Lobe	26 (29.2)	62 (28.2)	
Epencephalon	4 (4.5)	14 (6.4)	
Thalamus	2 (2.2)	4 (1.8)	
Brainstem	2 (2.2)	0 (0)	
Intraventricular hemorrhage, *n* (%)	41 (46.1)	116 (53.0)	0.272
Volume of hematoma, median (IQR)	44.38 (26.08–62.91)	47.63 (28.94–70.68)	0.651
**Body temperature profiles, median (IQR)**			
Body temperature at admission, (°C)	36.5 (36.5–36.7)	36.5 (36.5–36.7)	0.516
Maximum temperature within 24 h, (°C)	37.2 (36.9–37.4)	38.5 (38.0–39.0)	**<0.001**
Minimum temperature within 24 h, (°C)	36.2 (36.0–36.4)	36.9 (36.5–37.3)	**<0.001**
Fever burden, (°C × h)	0.00 (0.00–0.00)	7.00 (2.00–16.60)	**<0.001**
**Clinical outcomes**			
END, *n* (%)	19 (21.3)	63 (28.8)	0.182
3-month mRS score, median (IQR)	2 (2–5)	3 (2–5)	0.551
3-month poor functional outcome (mRS score > 2), *n* (%)	44 (49.4)	168 (54.5)	0.251

*p* value < 0.05 was shown in bold. END, early neurological deterioration; GCS, Glasgow coma score; ICH, intracerebral hemorrhage; IQR, interquartile range; mRS, modified Rankin Scale; WBC, white blood cell count.

**Figure 2:**
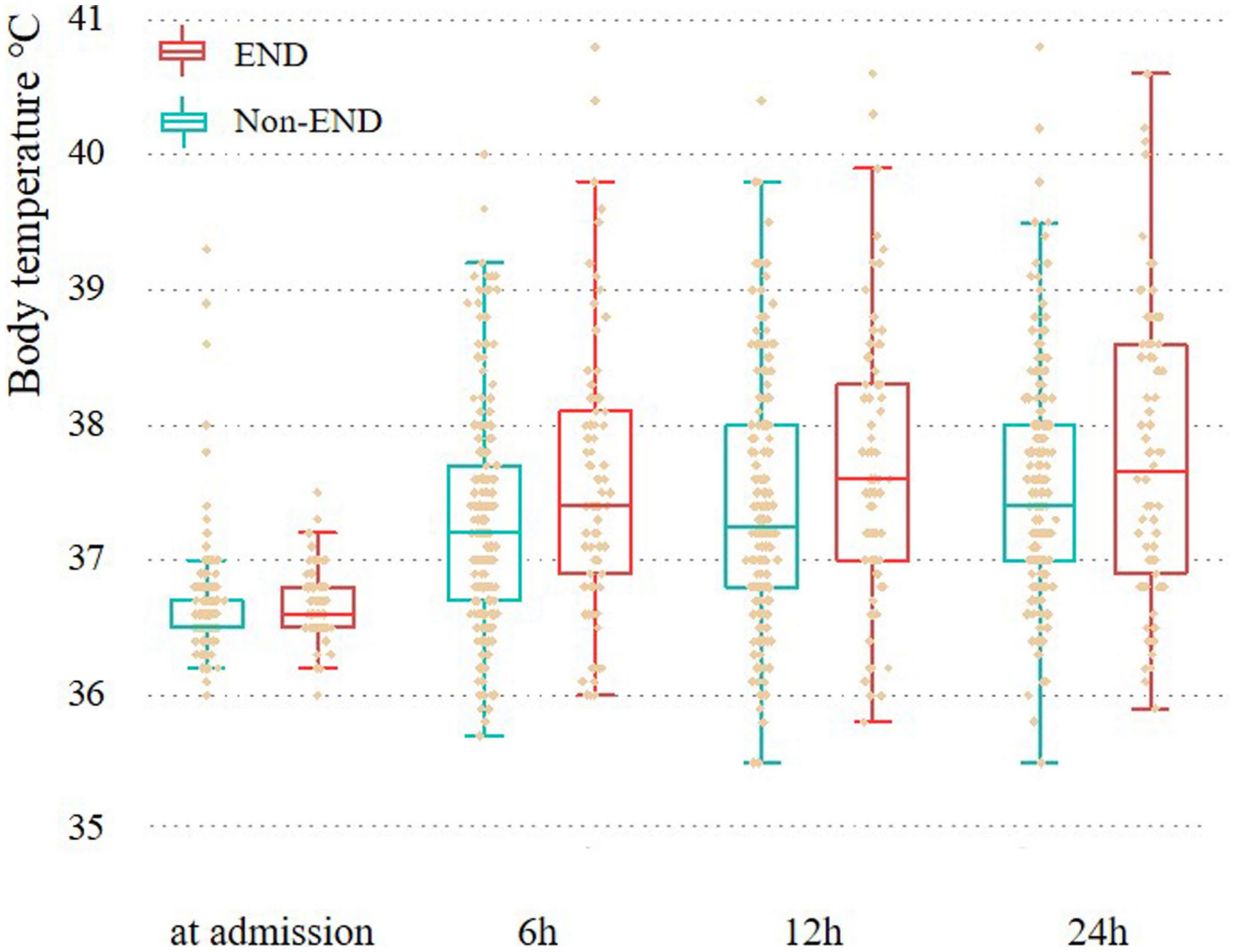
Body temperature at admission, 6 h, 12 h, and 24 h for patients with and without END. END, Early neurological deterioration.

**Figure 3:**
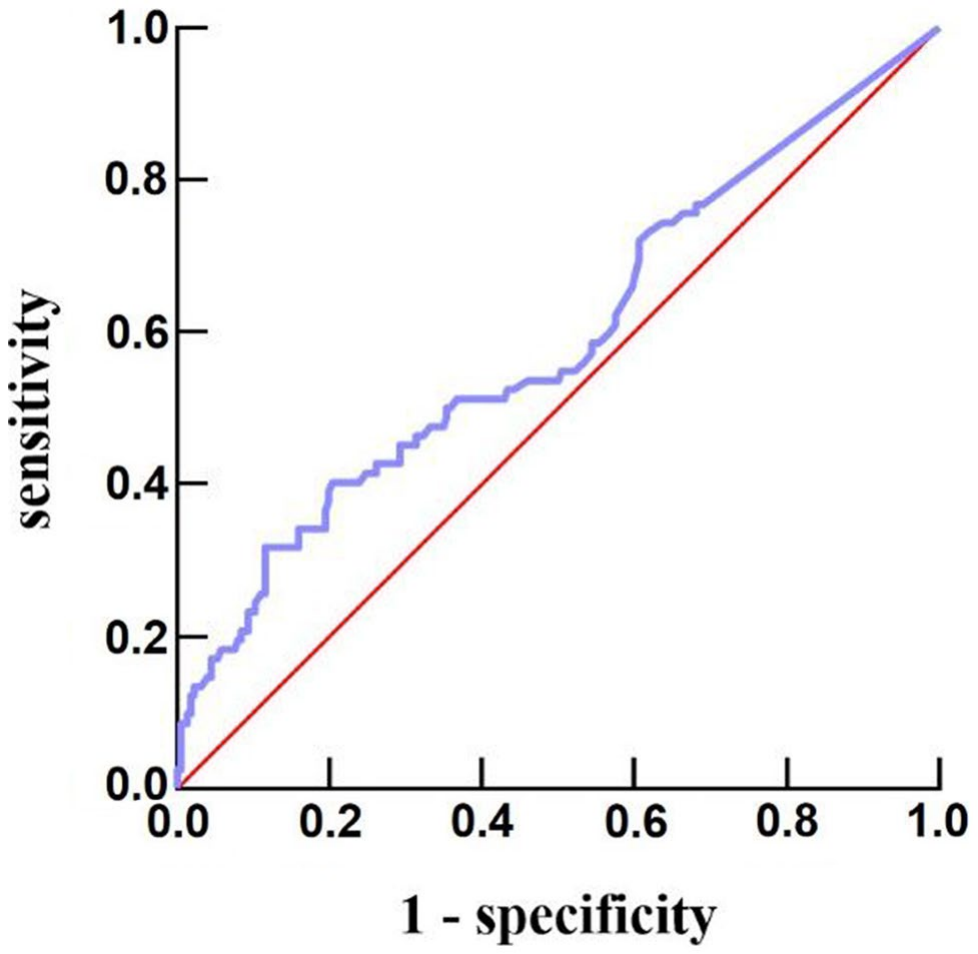
Predictive ability of fever burden under receiver operating characteristic curve for risk of END after acute intracerebral hemorrhage. END, early neurological deterioration.

The number of women (56 vs. 35, *p* = 0.016) and white blood cell count [8.80 (6.83–10.82) vs. 8.13 (6.19–10.29); *p* = 0.007] in the fever group were higher than those in the non-fever group, but age [51 (43–60) vs. 55 (50–64); *p* = 0.005] was lower. We plotted the body temperature distributions of patients with and without END at admission and at 6, 12, and 24 h after HE (As shown in Figure 2). In the multivariate logistic regression model, we adjusted for age (*p* = 0.553), sex (*p* = 0.041), systolic blood pressure at admission (*p* = 0.265), presence of hypertension (*p* = 0.944), hematoma volume (*p* = 0.872), and presence of intraventricular hemorrhage (*p* = 0.959) since these confounding factors were found to be associated with END in previous studies. As shown in Table 2, the burden of fever (OR = 1.055 per °C × h, 95% CI 1.008–1.103, *p* = 0.020) remained independently associated with END after adjusting for possible confounding factor. Fever burden was 12.17 ± 16.19 (median:5.2) in END group, and 6.29 ± 8.85 (median:2.45) in non-END group. ROC analysis was performed to determine the value of fever burden in predicting END. The cutoff value in the ROC curve (17.15°C × h) discriminated END (+)/END (−) patients with a sensitivity of 31.7%, specificity of 88.5%, PPV of 50%, and NPV of 78.1%. The area under ROC curve was 0.590 (95% CI, 0.514–0.666; Figure 3). Univariate logistic analysis showed that no factor was associated with poor functional outcomes 3 months after discharge (Supplementary Table S1).

**Table 2:** Results of multivariable logistic regression model to assess the relationship between body temperature indexes within 24 h after hematoma evacuation and early neurological deterioration.

	Crude OR (95% CI)	*p* value	Adjusted OR (95% CI)	*p* value
Body temperature 6 h after HE (per °C)	1.001 (0.609–1.889)	0.999	1.031 (0.576–1.848)	0.917
Body temperature 12 h after HE (per °C)	0.934 (0.547–1.562)	0.797	0.976 (0.565–1.687)	0.93
Body temperature 24 h after HE (per °C)	0.922 (0.555–1.570)	0.759	0.979 (0.576–1.663)	0.937
Maximum body temperature within 24 h (per °C)	0.860 (0.470–1.656)	0.636	0.802 (0.418–1.537)	0.506
Minimum body temperature within 24 h (per °C)	0.966 (0.459–1.872)	0.923	0.907 (0.440–1.871)	0.792
Duration of fever (per h)	1.008 (0.973–1.041)	0.633	1.007 (0.978–1.037)	0.631
Fever burden within 24 h (per °C × h)	1.053 (1.007–1.102)	**0.023**	1.055 (1.008–1.103)	**0.020**

*p* value < 0.05 was shown in bold. HE, hematoma evacuation; OR, odds ratio; Adjusted for age, sex, systolic pressure at admission, hypertension, hematoma volume, and intraventricular hemorrhage.

## 4. Discussion

END after ICH is associated with poor prognosis of death and major disability (7, 17). Therefore, it is important to identify the early clinical features of END. Previous studies have confirmed that blood pressure (18), uric acid (19), plasma leptin level (20), serum annexin A7 (21), and hematoma volume (17) can predict the occurrence of END after ICH. In this single-center retrospective study, END occurred in 26.6% patients. We found that fever burden was independently associated with the occurrence of END after HE in patients with ICH. Our results might help neurologists identify and deal with END promptly.

While hyperthermia is recognized as being associated with poor prognosis in patients with ICH (22, 23), discrepancies exist in the strategies for early postoperative temperature management following ICH (24, 25). Typically, a standard treatment regimen is employed in response to fever, encompassing both pharmacological and physical cooling measures. However, the effectiveness of these methods is hindered by the absence of a defined target temperature as the optimal temperature threshold remains unclear (26, 27). Our study provides a useful clinical insight: by monitoring the “fever burden” (the aggregate of the patient’s body temperature over time) within 24 h after surgery, clinicians may obtain an indicator for the potential occurrence of END.

The concept of fever burden was proposed in the 1990s and is defined as the product of fever and its duration. It has been used as a parameter to evaluate the effectiveness of cooling therapy in patients with neurological disorders (28). In previous studies, researchers found that body temperature > 37.5°C is an independent predictor of END (6). However, when the body temperature fluctuates greatly, or the peak temperature lasts for a prolonged period, the fever burden, which combines time and peak effects may better represent fever. Our results suggest that, when dealing with such patients, neurologists should pay attention not only to the highest fever temperature and/or duration of fever but also notice the combined indicators of the two aforementioned factors.

Infections have been associated with worse outcomes in patients with ICH (29) and might indirectly contribute to the onset of END by raising the patient’s body temperature. A retrospective study identified a link between infections in patients with ICH and neurological deterioration between 3 and 15 days post-ICH (30). In our study, most patients were admitted to the emergency department immediately following ICH onset and received emergent HE. Infections leading to fever within the first 24 h postoperatively were rare in this patient group. As a result, we did not include infections in our initial analysis. Future research is needed to investigate whether infections are associated with END within 24 h post-HE.

**Figure 4:**
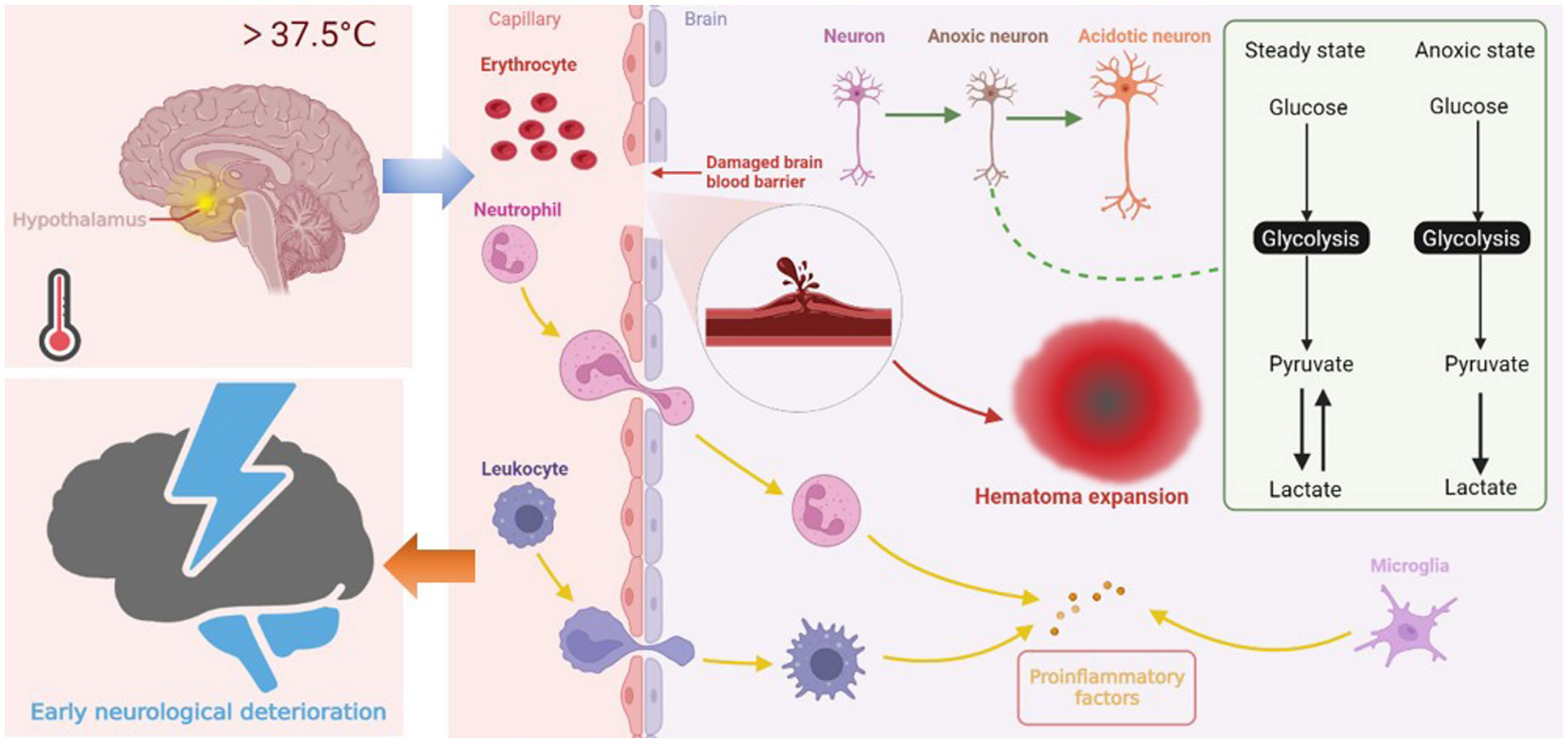
Central fever in the hypothalamus leads to END through three possible mechanisms. First, hyperthermia increases oxygen consumption of neurons, leading to compensatory glycolysis. Lactate accumulation and energy failure lead to cell edema and even necrosis. Second, fever affects blood-brain barrier permeability, increases edema around hematoma, creating conditions for hematoma expansion. Third, activated central immune cells and peripheral immune cells infiltrating through the damaged blood-brain barrier exacerbate the inflammatory response of cerebral hemorrhage, causing further damage to neurons.

Previous research has confirmed that the fever burden is associated with END after ischemic stroke (11). In patients with ICH, our study showed that fever burden is still associated with END after adjusting for potential confounding factors. A few mechanisms may explain our results. First, central fever regulated by the hypothalamus is the main cause of early fever after cerebral hemorrhage. Hyperthermia increases brain oxygen consumption and deprives neurons of energy reserves. Neurons are significantly vulnerable to hypoxia. Increased neuronal oxygen consumption results in compensatory glycolysis, which leads to lactate accumulation and cellular acidosis (22, 23). Energy failure and subsequent ATP-dependent ion channel dysfunction cause water to enter the cells, eventually leading to cellular swelling (24). Second, in addition to the direct effect of hyperthermia on brain cells, brain hyperthermia appears to affect the permeability of the blood-brain barrier (BBB) (25), which increases edema around the hematoma (26) and even causes hematoma expansion. Existing studies confirm hematoma expansion as one of the causes of END (1, 27, 29). Finally, a damaged BBB exacerbates the inflammatory response, leading to the activation of central and peripheral immune cells, which then release more molecules, including ROS, proteases and pro-inflammatory cytokines, leading to pathological immune reaction (30, 31). We plotted the possible mechanisms by which increased body temperature leads to END in Figure 4. However, the mechanism of END in ICH is heterogeneous, and further laboratory studies are warranted to determine the exact mechanism by which fever causes END.

Our study had several limitations. First, owing to the difficulty in implementing real-time temperature monitoring for each patient, body temperature was measured with a 6-h interval scheme, which means that the real maximum or minimum body temperature may not be collected. Therefore, future studies should focus on this issue. Second, our method of temperature measurement presents another limitation. Axillary temperature measurements, while convenient, may not accurately reflect core body temperature. This discrepancy can result in a less reliable measure of the patient’s true physiological state. To overcome this limitation, future studies should consider employing methodologies that allow for more accurate core body temperature monitoring. Third, the GCS is not widely used in the diagnosis of END, and future studies should perhaps use the NIHSS score to define END. Fourth, the area under the ROC curve of the fever burden in this study was relatively small (0.590) and the sensitivity is relatively low (31.7%), which may compromise the predictive value of the fever burden for END. Fifth, this study was conducted at a single-center with a limited sample size. The relatively small sample size may have compromised the power of our primary results. Finally, our study was conducted retrospectively; thus, any conclusions drawn are subject to the limitations of the respective study designs, including recall and observer bias. Future multicenter prospective studies are warranted to address these issues.

## 5. Conclusion

Fever burden is associated with END in patients with ICH undergoing HE. Our study provides more data on the relationship between the fever burden and the occurrence of early neurological deterioration in patients with intracerebral hemorrhage. Future studies with larger sample sizes are required to confirm these findings.

## Data availability statement

The original contributions presented in the study are included in the article/Supplementary material, further inquiries can be directed to the corresponding authors.

## Ethics statement

The studies involving human participants were reviewed and approved by the Ethics Committee of the Second Affiliated Hospital of Fujian Medical University. Written informed consent from the patients/participants or patients/participants’ legal guardian/next of kin was not required to participate in this study in accordance with the national legislation and the institutional requirements.

## Author contributions

H-fH, W-pH, and FZ conceived and designed the research. FW, S-lH, X-hW, and X-lC collected the data and performed the research. FW and YX processed and analyzed the data. All authors contributed to the article and approved the submitted version.
