# Supplementary material for: Fever burden within 24 h after hematoma evacuation predicts early neurological deterioration in patients with intracerebral hemorrhage: a retrospective analysis
Source: Front Neurol. 2023 Jul 19;14:1205031. doi: 10.3389/fneur.2023.1205031 (PMC10395082; doi:10.3389/fneur.2023.1205031)
Supplement: Supplementary file 2 [file Table_3.DOCX]

**Table 1** Results of univariable logistic regression model to assess the relationship between clinical signs of patients and poor functional outcome

|  | **p value** |
| --- | --- |
| Age | 0.247 |
| Sex | 0.231 |
| High blood pressure | 0.434 |
| Systolic blood pressure at admission | 0.411 |
| Diastolic blood pressure at admission | 0.455 |
| Heart rate at admission | 0.055 |
| Body temperature at admission | 0.639 |
| WBC | 0.763 |
| Serum glucose | 0.686 |
| Hematoma location | 0.736 |
| Volume of hematoma | 0.953 |
| [Intraventricular hemorrhage](javascript:;) | 0.758 |
| Body temperature 6 hours after HE | 0.770 |
| Body temperature 12 hours after HE | 0.987 |
| Body temperature 24 hours after HE | 0.781 |
| Maximum body temperature within 24 h | 0.827 |
| Minimum body temperature within 24 h | 0.869 |
| Duration of fever | 0.295 |
| Fever burden within 24 h | 0.634 |

HE, hematoma evacuation; WBC, white blood cell count.
